# Somatic and mental health of and the COVID-19 pandemic’s impact on adolescents diagnosed with asthma

**DOI:** 10.1038/s41533-025-00444-8

**Published:** 2025-08-11

**Authors:** Maria Emilsson, Kourosh Bador, Catrin Johansson, Nóra Kerekes

**Affiliations:** 1 Department of Health Sciences, University West, Trollhättan, Sweden; 2 Region Västra Götaland, Intensiv Care Unit, NU-Hospital Group, Trollhättan, Sweden; 3 Center for Holistic Psychiatry Research (CHoPy), Mölndal, Sweden

**Keywords:** Diseases, Health care

## Abstract

Adolescents with asthma are at heightened risk of somatic and mental health challenges, particularly during large-scale stressors such as the COVID-19 pandemic. This study explored the self-reported prevalence of asthma, co-occurring somatic complaints, psychological distress, and perceived pandemic impact in a multinational sample of 4802 upper secondary school students (aged 15–19) from Sweden, the United States, Serbia, Morocco, and Vietnam. Participants completed a web-based survey between November 2020 and June 2021. Approximately 9% reported having a physician-diagnosed asthma condition, with prevalence varying across countries. An additional 3.2% of the total sample reported uncertainty about whether they had asthma, with this uncertainty most frequently observed in Vietnam and Morocco. Adolescents with asthma reported slightly higher psychological distress than their peers without asthma, particularly among females, yet paradoxically reported a lower perceived impact of the pandemic on daily life. Physical activity levels were comparable between groups. Asthma was found to be associated with several co-occurring somatic complaints, with one gender-specific pattern observed in relation to thyroid disease. The observed variability in asthma prevalence and diagnostic uncertainty underscores the influence of national healthcare systems, health literacy, and communication practices. These findings highlight the need for gender-sensitive and context-aware approaches in adolescent health care, especially during global public health disruptions.

## Introduction

Asthma exists in people of all ages, and according to Global Initiative for Asthma^1^, it estimated to affect 300 million individuals worldwide^2^. It is the most common chronic condition in children (9.5%)^3,4^. During adolescence, there is a change in sex-specific risk and prevalence. While, in those under 13, asthma is more prevalent in males, the prevalence of asthma becomes higher in females during adolescence^5^. This change is suggested to be based on hormonal transformation and sex-specific environmental exposures^6^. It is estimated that 50% of adolescents and young adults with asthma-related symptoms are not receiving a medical diagnosis of asthma, mainly in low- and middle-income countries^1^.

Because asthma is a heterogeneous chronic respiratory disease, with the outbreak of COVID-19, it became important to establish whether people with asthma would be at increased risk of contracting or being seriously affected by another respiratory disease, SARS-CoV-2. While no increased risk of contracting COVID-19 or being seriously effected was shown^1^, nevertheless, people with asthma who required oral corticosteroids for their symptoms had an increased risk of death from COVID-19^1,7^.

A review of the past 20 years of research on asthma concluded in a comparison to the substantial body of research in children and adults that only a few studies have focused on adolescents, and the evidence level in this regard is therefore low^8^. Stress-related psychiatric disorders, such as depression and anxiety, are more common among adults with asthma^9,10^. Conversely, a higher prevalence of anxiety is often observed in adolescents with asthma, and this has been shown to affect their night-sleep negatively^11^. Disturbed night-sleep in children with asthma is related to night-time symptoms, such as nocturnal cough^12^. Disturbed night-sleep with shorter sleep duration has been reported in adolescents with asthma^13^. Interestingly, an increased prevalence of asthma is coupled with neuropsychiatric disorders, such as autism spectrum disorder and, most commonly, attention deficit/hyperactivity disorder (ADHD)^14,15^. Having any psychiatric disorder may result in increased asthma exacerbation and more emergency-room visits^8^.

According to a national survey in the US, 5% of children and adolescents with asthma have one or more coexisting health conditions, which is a greater prevalence than among children without asthma^16^. Adolescents with asthma have a greater risk of migraines as compared with those without asthma^17^. Several somatic diseases, which can each be coupled to systemic inflammation, such as chronic rhinitis, atopic dermatitis, urticaria, anaphylaxis, chronic sinusitis, irritable bowel syndrome, thyroid diseases, and allergies, are overrepresented among adolescents with asthma as compared to those without asthma^18^.

Importantly, to understand the impact of the high prevalence of co-existing conditions on children’s and adolescents’ quality of life, it should be considered that every additional chronic condition that occurs with asthma is associated with a greater likelihood of the need for medical attention, care and missed school days^16^. If the symptoms of asthma cannot be controlled despite relevant treatment, then there is a possibility that comorbidities are not being assessed and correctly treated, which leads to problems in asthma treatment^1^. In the present study, we collected self-reported data from adolescents from five countries during the first year of the COVID-19 pandemic. Respondents with and without self-reported diagnoses of asthma described their somatic and mental health and rated the overall impact of the pandemic on their lives. The present study utilized data from the multinational MeSHe survey to investigate health profiles and pandemic-related perceptions among adolescents with and without asthma. Specifically, the study aimed to assess: (1) the prevalence of allergic and non-allergic somatic comorbidities among adolescents with asthma; (2) their levels of psychological distress across multiple domains; and (3) how they perceived the overall impact of the COVID-19 pandemic on their daily lives. By identifying patterns across five diverse countries, this study contributes to a deeper understanding of how adolescents with chronic conditions navigate physical and psychological challenges, especially in the context of global disruptions like the COVID-19 pandemic.

## Methods

### Procedure

The current analysis is based on data from the Mental and Somatic Health without borders (MeSHe) epidemiological study (https://meshe.se/), a cross-sectional, multinational survey investigating adolescent health and well-being. The MeSHe study employed a stratified sampling method targeting upper-secondary school students aged 15–19 years across Sweden, Vietnam, Serbia, Morocco, and the US. Data collection occurred between September 2020 and February 2021 using paper-based and/or online survey methods, depending on country-specific logistics. Overall, 5114 adolescents participated, yielding a response rate of approximately 85% based on school attendance figures provided by participating institutions. A full description of the study protocol and procedures is available in Kerekes et al.^19^. The MeSHe survey, which was available in paper-and-pen and/or electronic versions, was translated into the participating countries’ languages and consisted of several validated questionnaires capturing adolescents’ self-rated mental and physical health; aggressive, antisocial, and self-harm behaviours; intensity and frequency of leisure time physical activity; mood; and personality. During this data collection, the MeSHe survey was completed along with a COVID-19-related questionnaire.

### Participants

The comprehensive MeSHe survey was completed by 5114 adolescents who were 15–19 years old (mean age 16.69, SD = 1.02). Table 1 summarizes the national and gender distribution of our study population. Asthma diagnosis was assessed through a self-report item asking whether the respondent had ever received a physician’s diagnosis of asthma. No additional questions about asthma symptoms or medication use were included in the survey. Although this self-report method aligns with international epidemiological approaches, we acknowledge the limitation of not being able to validate diagnoses with symptom data or clinical records. A total of 225 participants (4.4%) were excluded from the analysis due to missing or uncertain responses to the asthma question. Specifically, 64 did not respond and 161 selected “don’t know.” These individuals were excluded to ensure the validity of the comparison between clearly defined groups with and without asthma. There were also 82 respondents reporting a non-binary gender or not responding to the question concerning their gender identity. Excluding all the previously listed categories, the final study population in the present study consisted of 4807 adolescents.

**Table 1:** Gender distribution of the study population in the participating countries.

	Total N = 4807 (100%)	Women *n* = 3008 (62.6%)	Men *n* = 1799 (37.4%)	Age M = 16.69 (SD = 1.02)
Morocco	493 (10.3)	313 (63.5)	180 (36.5)	16.82 (1.22)
Serbia	1071 (22.3)	704 (65.7)	367 (34.3)	16.32 (1.06)
Sweden	1531 (31.8)	927 (60.5)	604 (39.5)	17.16 (0.87)
Vietnam	1417 (29.5)	840 (59.3)	577 (40.7)	16.45 (0.86)
USA	295 (6.1)	224 (75.9)	71 (24.1)	16.60 (0.99)

### Questionnaires

In the present study, the following instruments were utilized.

#### MeSHe health survey

The MeSHe health survey includes questions about the self-reported existence of somatic diseases and complaints. The survey has previously been used in adolescent populations^20,21^ and has shown good test-retest reliability^20^.

#### Brief Symptom Inventory (BSI)

The BSI measures an adolescent’s perceived mental distress and assesses it via 53 items in nine primary domains (somatization, obsession-compulsion, interpersonal sensitivity, depression, anxiety, hostility, phobic anxiety, paranoid ideation, and psychoticism) and a global index, the General Severity Index (GSI), which measures overall psychological distress levels. The BSI domains and its GSI have shown good reliability in multinational adolescent study samples, except for the hostility domain^22^; therefore, in the present study, we report scores for eight of the primary domains and the GSI.

#### The impact of the COVID-19 pandemic

In the COVID-19-related questionnaire, that was part of the MeSHe survey, there was one item that asked the respondent to rate, with a number between 0 (slight or no effect at all) and 10 (affected me immensely), the overall impact of the COVID-19 pandemic on their everyday life.

#### Godwin–Shephard leisure-time physical activity questionnaire (LTEQ)

The LTEQ is a 3 + 1-item scale for assessing the frequency and intensity of leisure-time physical exercise during a typical seven-day period^23^. The first three items measure how often the respondent engages in leisure-time physical activities of various intensities (mild, moderate, and strenuous). Based on the reported frequency of the workouts, the intensity of leisure-time exercise can be calculated mathematically (total leisure-time physical activity = 3 x mild + 5 x moderate + 9 x strenuous activities)^23^. The fourth item of the LTEQ is an independent question that measures the frequency of the various types of exercise that lasted longer than 15 min during the respondent’s leisure time. In the present study, we used the total leisure-time physical activity scores.

### Statistical analysis

The Statistical Package for the Social Sciences (SPSS) Version 28.0 was used for analyses. Descriptive statistics: frequencies (%), mean (M) and standard deviation (SD) were calculated. To compare asthma prevalence in adolescents between different country and the prevalence of somatic complaints/diagnoses between adolescents with and without asthma, Pearson’s Chi-square with risk estimate (odds Ratio) and Fisher’s exact tests were used, as well as Cramer’s V to report the effect size (small [0.10–0.29], medium [0.30–0.49] or large [≥ 0.500] with one degrees of freedom). Because the data on the scales were not normally distributed (*p* < 0.001 based on the Kolmogorov-Smirnov test), to compare the impact of COVID-19 and the level of psychological distress in adolescents with and without asthma, the Mann–Witney U-test was used, and η^2^ to report effect size (η^2^ = Z^2^/(N-1)) where η^2^ = 0.01 indicates a small effect; η^2^ = 0.06 indicates a medium effect; η^2^ = 0.14 indicates a large effect. To explore whether the relationship between asthma and comorbid somatic conditions varied by gender, interaction terms (condition × gender) were included in the logistic regression models. Only participants identifying as male or female were included in these analyses. Internal consistency of the psychometric scales used in the study was evaluated using Cronbach’s alpha. Across the instruments used, Cronbach’s alpha ranged from 0.73 to 0.97, indicating acceptable to excellent reliability. These values apply to the eight domains and the global index (GSI) of the Brief Symptom Inventory (BSI).

## Results

### The prevalence of asthma in a multinational sample of adolescents

Approximately 9% (*n* = 448) of the respondents, a similar proportion of both genders, reported having received a doctor-diagnosed asthma diagnosis. There were 3.2% (*n* = 161) who did not know whether they had asthma or not. The distribution of the asthma cases and those with indefinite responses is provided according to nationality in Table 2.

**Table 2:** The prevalence of adolescents with an asthma diagnosis in the multinational study population by country.

	Prevalence of a diagnosis of asthma % (n)			Prevalence of adolescents indicating uncertainty about whether they have asthma % (n)		
	Total	Women	Men	Total	Women	Men
Study population	9.0 (448)	9.0 (280)	9.0 (168)	3.2 (161)	3.2 (99)	3.3 (62)
Morocco	5.9 (29)	5.4 (17)	6.7 (12)	5.2 (27)	5.2 (17)	5.3 (10)
Serbia	7.0 (75)	7.0 (49)	7.1 (26)	1.1 (12)	0.8 (6)	1.6 (6)
Sweden	16.7 (256)	16.4 (156)	16.0 (100)	3.0 (47)	2.8 (27)	3.2 (20)
Vietnam	2.8 (39)	2.1 (18)	3.6 (21)	4.5 (67)	4.8 (42)	4.2 (25)
The US	16.6 (49)	17.9 (40)	12.7 (9)	2.6 (8)	3.0 (7)	1.4 (1)

The study population *n* = 4802; 3007 women and 1795 men.

The differences in the prevalence of asthma and in the proportion of adolescents who did not know if they have asthma or not was significant, with a small effect size (Cramer’s V = 0.153, *p* < 0.001) between countries. In the US and Sweden, considerably more and, in Vietnam and Morocco, considerably fewer adolescents reported asthma, while in Vietnam and Morocco, considerably more and, in Serbia, considerably fewer adolescents reported that they were unsure whether they had asthma than was statistically expected.

### The prevalence of somatic complaints in adolescents with and without asthma

Table 3 summarizes the prevalence of defined somatic diseases and gastrointestinal complaints in the study population. The prevalence of each somatic disease, except cancer, diabetes, epilepsy, and skin diseases, and the two assessed gastrointestinal complaints (constipation and diarrhoea) were significantly more frequently reported in adolescents with asthma as compared to those reporting no asthma. The odds of an adolescent who had asthma also reporting the existence of other allergies was increased 10-fold as compared to an adolescent who had no asthma. The co-occurrence of allergies with asthma showed a moderately strong difference in effect as compared to the prevalence of allergies in adolescents without asthma (Cramer’s V = 0.36). There was a high odds ratio for with asthma coexisting with tuberculosis; however, the effect size of this association was negligible due to the low prevalence of tuberculosis in our study population. The odds of an adolescent with asthma also suffering from other inflammatory/autoimmune diseases, such as celiac disease, thyroid diseases, or rheumatologic disease, were 2–3-fold higher than those of an adolescent without asthma, and the odds of asthma were increased by 70% in cases of migraine, however, each of these had a negligible effect size (data not shown).

**Table 3:** Prevalence of somatic diseases and complaints in the groups of adolescents with and without asthma.

	Comparison group % (n)	Asthma group % (n)	*p*-value*	OR	95% CI**
Allergy	19.0 (4052)	70.4 (432)	< 0.001	10.11	8.04–12.80
Cancer	0.5 (4321)	1.1 (452)	0.11	2.19	0.52–5.03
Celiac	1.1 (4221)	2.7 (437)	0.005	2.46	1.22–4.29
Diabetes	0.7 (4334)	1.1 (448)	0.28	1.6	0.37–3.72
Epilepsy	0.8 (4329)	1.1 (450)	0.47	1.42	0.34–3.08
Migraine	8.2 (4100)	13.4 (426)	< 0.001	1.74	1.26–2.27
Rheumatologic disease	1.0 (4255)	2.8 (435)	0.001	2.78	1.18–5.08
Skin disease	10.8 (4182)	13.2 (441)	0.13	1.25	0.91–1.64
Thyroid disease	1.2 (4260)	2.3 (432)	0.038	2.04	0.80–3.78
Tuberculosis	0.1 (4357)	1.8 (445)	< 0.001	11.59	3.47–46.38
Constipation	7.0 (4051)	12.8 (415)	< 0.001	1.94	1.40–2.52
Diarrhoea	4.1 (4019)	7.6 (406)	0.001	1.92	1.27–2.74

*OR* odds ratio after 1000-interation simple bootstrapping.

*significance level with bootstrap-coupled Pearson’s Chi-square test; **Bias corrected and accelerated confidence interval (CI).

In the initial logistic regression models testing the association between asthma and various somatic conditions, gender was included as a covariate. Across these models, gender alone did not significantly predict asthma status. However, a significant interaction was found between gender and thyroid disease (*p* = 0.017), indicating a differential association by gender. To further explore this, we conducted stratified analyses by gender. As shown in Table 4, adolescent men with asthma had notably higher odds of reporting certain co-occurring somatic conditions and gastrointestinal complaints compared to adolescent women with asthma. These differences were especially pronounced for cancer, diabetes, thyroid disease, tuberculosis, and constipation, where odds ratios in adolescent men were several times greater than those observed in their female counterparts.

**Table 4:** Prevalence of somatic diseases and complaints in adolescent men and adolescent women with and without asthma.

		Comparison group	Asthma group	*P*-value*	OR	95% CI**
Allergy	Women	20.0% (496)	71.6% (187)	< 0.001	10.10	7.65–13.69
	Men	17.2% (262)	70.3% (111)	< 0.001	11.33	7.85–16.76
Cancer	Women	0.6% (15)	0.4% (1)	0.67	0.64	0.43–2.92
	Men	0.4% (7)	2.5% (4)	0.002	5.80	1.19–19.68
Celiac disease	Women	1.3% (35)	3.0% (8)	0.037	2.24	0.87–3.94
	Men	0.8% (13)	1.9% (3)	0.17	2.37	0.64–6.60
Diabetes	Women	0.6% (17)	0.4% (1)	0.57	0.57	0.41–2.43
	Men	0.7% (12)	1.9% (3)	0.14	2.53	0.63–8.24
Epilepsy	Women	0.6% (15)	0.7% (2)	0.73	1.30	0.48–3.93
	Men	1.1% (18)	1.3% (2)	0.89	1.1	0.41–3.69
Migraine	Women	9.2% (231)	14.8% (38)	0.004	1.71	1.16–2.45
	Men	6.6% (101)	10.4% (16)	0.073	1.65	0.85–2.63
Rheumatism	Women	1.1% (29)	2.6% (7)	0.032	2.43	0.82–5.36
	Men	0.9% (14)	3.2% (5)	0.008	3.71	0.76–9.56
Skin disease	Women	11.5% (296)	15.4% (41)	0.06	1.40	0.94–2.01
	Men	9.4% (147)	10.6% (17)	0.64	1.14	0.62–1.83
Thyroid disease	Women	1.6% (41)	1.9% (5)	0.68	1.22	0.28–2.60
	Men	0.4% (7)	3.2% (5)	< 0.001	7.41	1.70–25.82
Tuberculosis	Women	0.1% (4)	1.5% (4)	< 0.001	10.01	2.13–47.23
	Men	0.1% (2)	1.9% (3)	< 0.001	15.27	3.06–61.67
Constipation	Women	9.0% (223)	12.9% (33)	0.038	1.51	0.98–2.12
	Men	3.7% (56)	11.5% (17)	< 0.001	3.37	1.68–5.76
Diarrhea	Women	3.9% (96)	6.0% (15)	0.100	1.59	0.79–2.64
	Men	4.7% (70)	8.3% (12)	0.060	1.83	0.96–3.11

*OR* odds ratio after 1000-interation simple bootstrapping.

*significance level with bootstrap-coupled Pearson’s Chi-square test; **Bias corrected and accelerated confidence interval (CI).

### The level of psychological distress in adolescents with and without asthma

Adolescents with asthma reported a significantly higher distress level in each domain of the BSI and on the GSI as compared to adolescents without asthma, but with a negligible effect size, except for somatization, for which the effect size was small (Table 5).

**Table 5:** The level of psychological distress in adolescents with and without asthma.

	Comparison group *n* = 3471–3943	Asthma group *n* = 363–413	*P*	η^2^
	Mean (SD)	Mean (SD)		
Anxiety	1.07 (0.94)	1.34 (1.00)	< 0.001	0.007
Depression	1.35 (1.07)	1.62 (1.11)	< 0.001	0.005
Interpersonal Sensitivity or Social Insecurity	1.33 (1.09)	1.51 (1.12)	< 0.001	0.002
Obsessive-Compulsive Behavior	1.41 (0.98)	1.73 (1.02)	< 0.001	0.008
Paranoid Ideation	1.14 (0.96)	1.33 (1.01)	< 0.001	0.003
Psychoticism	0.96 (0.90)	1.11 (0.95)	< 0.001	0.003
Phobia Anxiety	0.84 (0.85)	1.01(1.01)	0.006	0.002
Somatization	0.82 (0.80)	1.15 (0.90)	< 0.001	0.014
GSI (without Hostility)	1.11 (0.81)	1.37 (0.85)	< 0.001	0.008

Comparison group, those reporting no asthma.

*GSI* general severity index.

When analyzing psychological distress levels in adolescent men and adolescent women with and without asthma, it appeared that the above-described differences were enlarged in adolescent women, while the difference in the level of phobic anxiety in adolescent men with and without asthma did not differ significantly (Table 6). Adolescent women with asthma reported a significantly but with negligible effect size increased levels of psychological distress in all domains and on the GSI than adolescent men with asthma (Table 6).

**Table 6:** Psychological distress in adolescent men and adolescent women with and without asthma.

		Comparison group		Asthma group	*P*		*P*
		*n* = 3471– 3943		*n* = 363–413	Between genders in the Asthma Group	η^2^	Between Comparison Group and Asthma Group
		Mean (SD)		Mean (SD)			
Anxiety	Women	1.25 (0.96)	1.55 (1.01)	< 0.001	0.008		< 0.001
	Men	0.76 (0.82)	1.01 (0.86)				< 0.001
Depression	Women	1.49 (1.07)		1.74 (1.09)	0.004	0.002	< 0.001
	Men	1.11 (1.03)		1.26 (0.96)			< 0.001
Interpersonal Sensitivity/Social Insecurity	Women	1.52 (1.10)		1.71 (1.12)	< 0.001	0.006	0.011
	Men	0.99 (0.99)		1.20 (1.05)			0.044
Obsessive-Compulsive Behavior	Women	1.57 (0.98)		1.88 (1.01)	< 0.001	0.004	< 0.001
	Men	1.14 (0.90)		1.36 (0.84)			< 0.001
Paranoid Ideation	Women	1.26 (0.96)		1.45 (1.01)	< 0.001	0.003	0.002
	Men	0.92 (0.92)		1.15 (0.84)			0.019
Psychoticism	Women	1.06 (0.91)		1.20 (0.94)	0.009	0.002	0.013
	Men	0.81 (0.85)		1.00 (0.93)			0.017
Phobia Anxiety	Women	0.99 (0.88)		1.24 (1.01)	< 0.001	0.012	< 0.001
	Men	0.59 (0.71)		0.70 (0.71)			0.69
Somatization	Women	0.96 (0.83)		1.34 (0.93)	< 0.001	0.007	< 0.001
	Men	0.58 (0.67)		0.76 (0.78)			< 0.001
GSI (without Hostility)	Women	1.26 (0.82)		1.51 (0.84)	< 0.001	0.005	< 0.001
	Men	0.85 (0.73)		1.11 (0.77)			< 0.001

Comparison group, those reporting no asthma.

*GSI* general severity index.

### Leisure-time physical activity

The total score on the LTEQ was somewhat but not significantly (*p* = 0.21) lower in adolescents with asthma (M = 49.27; SD = 42.13, Md = 39) than in those without asthma (M = 52.10; SD = 43.77, Md = 43). There were no significant differences if the analysis was performed within the groups of adolescent men (*p* = 0.57) or adolescent women (*p* = 0.22).

### The COVID-19 pandemic’s impact on adolescents with and without asthma

Adolescents reported the COVID-19 pandemic’s impact on their everyday lives on a scale between 0 and 10. The mean score for the study population was 4.46 (SD = 2.93). Adolescents with asthma reported a significantly (*p* = 0.026) lower impact (M = 4.16, SD = 2.90) on the part of the pandemic on their lives than adolescent without asthma (M = 4.47, SD = 2.92). There was no significant difference in the self-rated impact of the COVID-19 pandemic on adolescent women and adolescent men with asthma (*p* = 0.95); however, adolescent women with asthma reported a significantly (*p* = 0.009) lower impact (M = 4.19, SD = 2.90) on the part of the pandemic on their everyday lives than their same sex counterparts without asthma (M = 4.64, SD = 2.88). There was no significant difference between adolescent men with and without asthma concerning the impact of the COVID-19 pandemic on their everyday lives.

## Discussion

The MeSHe project made it possible to assess the prevalence of somatic and mental health complaints and the impact of the COVID-19 pandemic in a multinational sample of adolescents with and without asthma. The prevalence of asthma, based on self-reports, was about 9%, with a similar prevalence having been reported in men and women, 15–19-year-old upper secondary school students at the end of 2020. The International Study of Asthma and Allergies in Children (ISAAC)^24^ indicated a comparably higher prevalence (13.0% and 14.6%) of asthma for 13–14-years-old men and women, respectively, in 2013. The prevalence of physician-diagnosed asthma in men aged 16 to 18 years old has been reported to be 11.4%^25^. The findings of this study reveal the highest prevalence in the adolescent multinational population was measured in Sweden and the US, and the lowest was measured in Vietnam. Wide variability in the prevalence of asthma has been previously identified not only between countries but also between regions in same country and even between different centres in the same city^24^. The reason for these variations is likely the complex combined effect of bio-psycho-social factors. It has been previously suggested that the prevalence of asthma in developing countries is increasing^2^. However, even in this context, there is no consensus in the research field^26^. In our multinational sample, the highest prevalence was indeed found in the two most developed countries (the US and Sweden, with both having prevalence rates around 16%). However, it is important to recognize that, in these countries, there was also the lowest rate of uncertainty about having asthma. The highest rate of those who did not know whether they have asthma or not was reported from Morocco, followed by Vietnam. This observation—of adolescents reporting uncertainty about whether they have received an asthma diagnosis—may reflect broader challenges related to health literacy, diagnostic communication, and access to health care. In contexts where healthcare systems are under-resourced or where disease awareness is lower, young people may be less likely to receive timely, clear information about chronic conditions like asthma. A recent study of Swedish adolescents with ADHD highlighted a related issue: adolescents who experienced greater uncertainty about their health status also reported increased somatic complaints and anxiety regarding undiagnosed conditions^27^. While our study does not focus on adolescents with ADHD, the parallel finding of diagnostic uncertainty among adolescents in countries like Vietnam and Morocco may suggest a shared vulnerability in health-related understanding. This underscores the need to support adolescents’ health literacy and ensure that diagnoses are clearly communicated across diverse healthcare settings.

Because the MeSHe survey did not measure socioeconomic status or family-related biological factors, we can only speculate regarding the reason for this significantly higher prevalence of asthma in developed countries. Our hypothesis is that the high quality of healthcare ensures widespread knowledge in such societies about the symptoms of asthma and enables the early identification of the disease.

Adolescents with asthma had significantly higher odds of having several somatic diseases and/or gastrointestinal complains. However, the difference in odds ratios was accompanied by a small or negligible effect size, except for the odds ratio for co-existing allergies. Allergies are the most common comorbidity, which aligns with the clinical phenotype of allergic asthma, which is triggered by allergens and classified as type 2 inflammation^1^. A similar result was found in a study from Italy involving children and adolescents, in which the clinical significance of type 2 inflammation in asthma pathology and its implications for the treatment of asthma were highlighted^28^. A better comprehension of the underlying mechanisms of asthma may contribute to our understanding of the disease and its association with various comorbidities, such as allergies.

In our study, adolescents with asthma reported a significantly higher prevalence of celiac disease and rheumatoid arthritis as compared to those without asthma. Our results corroborate a previous study^29^ that similarly demonstrated a higher likelihood of adolescents with celiac diseases also having asthma. Also, individuals with asthma have a significantly elevated risk of developing rheumatoid arthritis in comparison to those without asthma^30^. While these findings are aligned with our expectations, it is important to note that, in our study, although these differences were statistically significant, none of them reached the threshold for a small effect size. Therefore, they should be referred to with caution.

As As discussed above, both adolescent males and females with asthma demonstrated increased odds of reporting various co-occurring somatic conditions and gastrointestinal complaints. However, most of these associations were characterized by small effect sizes. Among adolescent males with asthma, the associations with thyroid disease, tuberculosis, and constipation reached statistical significance and showed small but meaningful effect sizes when compared to males without asthma. These results align with previous findings indicating that adolescent males with asthma commonly present with comorbidities such as chronic rhinitis, atopic dermatitis, urticaria, anaphylaxis, irritable bowel syndrome, and thyroid disorders^18^. Notably, that study did not report a higher prevalence of rheumatism, consistent with our own observations. Our analyses also revealed a significant interaction between gender and thyroid disease, suggesting that the relationship between asthma and thyroid disease differed by gender. Specifically, adolescent females with thyroid disease were more likely than their male counterparts to report asthma, which aligns with existing knowledge about gender differences in autoimmune and endocrine conditions. No other gender-by-condition interactions reached statistical significance. These findings underscore the importance of interpreting statistically significant results in the context of effect sizes and sample distribution and highlight the complexity of asthma-related comorbidities. They also point to the value of incorporating gender-sensitive perspectives in adolescent health research and care planning.

Adolescents with asthma exhibited a statistically significantly, albeit negligibly, higher level of psychological distress during the COVID-19 pandemic as compared to their counterparts without asthma. This discrepancy was particularly noticeable among adolescent women, who reported significantly higher levels of psychological distress than their men peers with asthma. It is noteworthy that adolescent women, in general, tend to exhibit significantly higher levels of psychological distress^19,22,31^. Thus, it is premature to conclude that the COVID-19 pandemic placed any elevated psychological burden on adolescents with asthma. Surprisingly, these individuals, mainly adolescent women, reported a significantly lower impact on the part of COVID-19 as compared to their peers without asthma. This may be attributed to their limited exposure to severe COVID-19 cases given their age and asthma. In a Swedish asthma population under the age of 18, only 0.02% (eight out of 46,123 patients) required hospitalization due to COVID-19^32^. Similarly, in the US, among 5656 children aged 5 to 18, there were no COVID-19-related hospitalizations^33^. During the pandemic years, as stated by GINA^1^, the consistent use of asthma medication remained crucial. Notably, during these years, there was a significant reduction in asthma exacerbation^33^. Concurrently, visits to pediatric emergency departments due to asthma showed a significant decline^34^. Maintaining optimal asthma control minimizes the risk of exacerbation, subsequently reducing the likelihood of hospitalization and lowering COVID-19 exposure^35^. The benefits of adhering to inhaled corticoid steroid treatment clearly outweigh the potential risks of severe COVID-19. As a result, recommendations endorse the utilization of inhaled corticoid steroid in individuals with asthma^35,36^.

Based on our findings, another potential protective factor working against the adverse psychological impact of the COVID-19 pandemic on adolescents with asthma is their level of leisure-time physical activity, which is comparable to that of their peers without asthma. In contrast, a few years ago, research suggested that adolescents with asthma exhibited lower levels of physical activity as compared to those without asthma^37^. The current findings could reflect the positive outcomes of enhanced asthma care, in which structured education emphasizes the significance of engaging in physical activity^1^. Regular physical activity offers important health benefits and can positively impact asthma control and lung function^1^. Moreover, evidence indicates that physical activity is associated with reduced psychological distress^38^. This dual benefit highlights the potential role of physical activity in mitigating both the physical and psychological challenges posed by asthma, particularly in the context of the COVID-19 pandemic. In addition to individual-level factors, the broader national context may have influenced how adolescents with asthma perceived the pandemic’s impact. Our study included participants from five countries with varying public health strategies. For instance, Sweden did not impose formal lockdowns, while Vietnam and Serbia implemented stricter restrictions. These policy differences may have shaped adolescents’ day-to-day experiences during the pandemic. Although both groups—those with and without asthma—were drawn from the same upper secondary high-school populations within each country, and were thus similarly exposed to national conditions, the overall comparison must still be interpreted with caution. It is plausible that the milder reported COVID-19 impact among adolescents with asthma reflects both effective asthma management and contextual factors such as reduced social disruption in certain countries. Taken together, these findings suggest that both effective asthma self-management and national-level public health strategies may have played a role in buffering the perceived impact of the COVID-19 pandemic among adolescents with asthma, offering new insights into resilience factors in youth with chronic conditions.

### Strengths and limitations

This study offers several strengths that enhance its contribution to the understanding of adolescent health during the COVID-19 pandemic. First, its multinational scope—encompassing upper-secondary students from five culturally and geographically diverse countries—enables a broad perspective on adolescent well-being across contexts. The sample size is substantial, and the inclusion of both adolescents with and without asthma allowed for comparative analyses across key physical and psychological domains. Second, the study collected data during the first year of the pandemic, providing timely insight into how adolescents perceived and responded to an unprecedented global health crisis. Third, the dataset uniquely enabled a comprehensive examination of coexisting somatic conditions, psychological distress, and physical activity levels among adolescents with asthma, contributing to the limited literature on chronic illness in youth during times of societal stress.

Despite these strengths, several limitations must be acknowledged. One notable limitation is the study’s reliance on self-reported data, including asthma diagnosis and psychological symptoms. While self-report methods are common and practical in large-scale adolescent research, they may introduce recall bias or inaccuracies in diagnostic reporting. The relatively high proportion of adolescents who indicated uncertainty about whether they had asthma further underscores this point, highlighting that the findings are not based on clinically verified diagnoses. In addition, the study did not include measures of asthma severity or control, which would have allowed for more nuanced interpretation of health outcomes within the asthma subgroup.

The cross-sectional design is another limitation, as it precludes any causal conclusions. All associations observed—between asthma status, comorbidities, psychological distress, and the perceived impact of the pandemic—are correlational and should be interpreted accordingly.

Moreover, while we reported asthma prevalence at the country level, we refrained from conducting stratified country-specific analyses due to the non-representative nature of national subsamples. As such, findings are best understood in relation to the overall multinational sample and should not be generalized to specific national populations.

Lastly, national context is an important consideration. The five participating countries implemented markedly different public health strategies during the pandemic. For example, Sweden did not enforce formal lockdowns, while Vietnam and Serbia applied stricter containment measures. These differences may have influenced how adolescents perceived the pandemic’s impact. Although both groups—those with and without asthma—were drawn from the same school populations within each country, and thus likely experienced similar contextual exposures, we acknowledge that country-level policy environments could have affected self-reported outcomes. We now address this more explicitly in the Discussion, urging caution in interpreting the results without considering the broader societal context.

## Conclusion

This multinational study offers important insights into the somatic and mental health of adolescents with asthma during the COVID-19 pandemic. Adolescents with asthma reported slightly higher levels of psychological distress than their peers without asthma, particularly among females. However, the overall impact of the pandemic on daily life was perceived as lower in the asthma group. Physical activity levels were comparable between groups, suggesting potential resilience factors among adolescents managing chronic illness.

The results also highlight significant variability in self-reported asthma prevalence and diagnostic certainty across countries, pointing to differences in health literacy, diagnostic practices, and healthcare access. Notably, the higher proportion of adolescents in Vietnam and Morocco who were unsure about their asthma diagnosis underlines the importance of clear health communication and supports the need for improved adolescent health literacy.

While the findings must be interpreted in light of the study’s cross-sectional design and non-representative national samples, they underscore the complexity of asthma’s psychosocial context in adolescence. They also emphasize the value of cross-national perspectives in understanding how public health infrastructure and cultural factors may shape young people’s experiences of chronic illness, particularly during global disruptions such as the COVID-19 pandemic.

## Data Availability

Data will be made available on reasonable request to the project leader, Professor Nóra Kerekes (nora.kerekes@hv.se).

## Content for publications

All authors have read and agreed to the published version of the manuscript.
